# CLASSIFICATION OF UPPER LIMB SPASTICITY PATTERNS IN PATIENTS WITH MULTIPLE SCLEROSIS: A PILOT OBSERVATIONAL STUDY

**DOI:** 10.2340/jrm.v56.40548

**Published:** 2024-08-25

**Authors:** Mirko FILIPPETTI, Linde LUGOBONI, Rita DI CENSO, Luca DEGLI ESPOSTI, Salvatore FACCIORUSSO, Valentina VARALTA, Andrea SANTAMATO, Massimiliano CALABRESE, Nicola SMANIA, Alessandro PICELLI

**Affiliations:** 1 Neuromotor and Cognitive Rehabilitation Research Center, Section of Physical and Rehabilitation Medicine, Department of Neuroscience, Biomedicine, and Movement Sciences, University of Verona, Verona, Italy; 2 Canadian Advances in Neuro-Orthopedics for Spasticity Consortium (CANOSC), Kingston, ON, Canada; 3 Neurorehabilitation Unit, Department of Neurosciences, University Hospital of Verona, Italy; 4 Spasticity and Movement Disorders “ReSTaRt” Unit, Physical Medicine and Rehabilitation Section, Riuniti Hospital, University of Foggia, Foggia, Italy; 5 Neurology Section, Department of Neurosciences, Biomedicine and Movement Sciences, University of Verona, Verona, Italy

**Keywords:** botulinum toxins, multiple sclerosis, muscle spasticity, symptom assessment, upper extremity

## Abstract

**Objective:**

The aim of this study was to provide a classification of the upper limb patterns in patients with upper limb spasticity due to multiple sclerosis.

**Design:**

Pilot observational study.

**Patients:**

Twenty-five adult patients with multiple sclerosis suffering from upper limb spasticity who underwent one segmental (i.e., proximal and distal upper limb) botulinum toxin treatment cycle were recruited.

**Methods:**

Patients remained in a sitting position during the evaluation. Upper limb spasticity postures (i.e., postural attitude of a single joint/anatomical region) were evaluated and recorded for the shoulder (adducted/internally rotated), elbow (flexed/extended), forearm (pronated/supinated/neutral), wrist (flexed/extended/neutral) and hand (fingers flexed/thumb in palm).

**Results:**

On the basis of the clinical observations, 6 patterns (i.e., sets of limb postures) of upper limb spasticity have been described according to the postures of the shoulder, elbow, forearm, and wrist.

**Conclusion:**

The patterns of upper limb spasticity in patients with multiple sclerosis described by this pilot study do not completely overlap with those observed in patients with post-stroke spasticity. This further supports the need to consider the features of spasticity related to its aetiology in order to manage patients appropriately.

## LAY ABSTRACT

This pilot study aimed to describe typical positions of the arm in people with multiple sclerosis suffering from muscle overactivity. We included 25 adults who were treated once with botulinum toxin injection into the arm muscles. After clinical evaluation we defined 6 sets of combined positions (the so-called patterns) involving the shoulder, elbow, forearm, and wrist. These patterns were peculiar to people with multiple sclerosis with only few similarities to individuals with stroke. Outlining the specific presentation in patients with multiple sclerosis will help to provide more appropriate clinical support, such as defining proper injection regimens of botulinum toxin type A, which is a first-line treatment for muscle overactivity.

Multiple sclerosis (MS) is a chronic inflammatory disease of the central nervous system characterized by demyelination and axonal loss (1). It represents a main cause of neurological disability in young adults (2). The 66% of patients with MS suffer from sensorimotor impairments of the upper limbs, which considerably impact some activities of daily living (3). Spasticity is a major disabling symptom in MS, which affects up to 80% of patients during the time course of disease as a consequence of lesions involving the descending pathways to the spinal motor circuits (i.e., corticospinal and dorsal reticulospinal tracts) (4).

Two-thirds of patients with MS who suffer from spasticity identify it as a top priority for treatment (5). Botulinum toxin type-A (BoNT-A) is a first-line treatment for focal spasticity in patients with MS (6, 7). Guidelines recommend carefully defining the clinical presentation of spastic limbs to appropriately select the overactive muscles for BoNT-A injection (8). In this context, upper limb spasticity postures and patterns have been classified only in patients with stroke by Hefter and colleagues (9). However, even though upper limb spasticity postures (i.e., postural attitude of a single joint/anatomical region) may be similar in stroke and other aetiologies of upper motor neurone syndrome (e.g., adducted and internally rotated shoulder, flexed elbow, pronated forearm, and flexed wrist), it is conceivable that patients with MS might present with peculiar spasticity patterns (i.e., sets of limb postures) due to the heterogenicity of demyelination lesion locations, which impacts the clinical course of the disease (10).

To the best of our knowledge, the patterns of upper limb spasticity have not yet been defined in patients with MS. Therefore, the primary aim of this pilot study was to provide a classification of upper limb patterns based on the description of upper limb postures in patients with MS suffering from spasticity. Our secondary aim was to report retrospectively on BoNT-A treatment of upper limb spasticity due to MS according to its clinical presentation (i.e., postures and patterns).

## METHODS

This was a single-centre, pilot, observational study. Eligibility criteria were as follows: confirmed diagnosis of MS; age greater than 18 years; upper limb spasticity that previously underwent 1 segmental (i.e., proximal and distal upper limb) BoNT-A treatment cycle. Non-eligibility criteria were: bony deformities in the upper limbs; previous treatments of upper limb spasticity with neurolytic or surgical procedures; other neurological or orthopaedic conditions involving the upper limbs. All participants were outpatients referred to our clinical unit to be screened for project *2022/R-Single/031*, which was funded by the Fondazione Italiana Sclerosi Multipla (FISM) and approved (number 4257CESC) by the local Ethical Committee (Comitato Etico Territoriale Area Sud-Ovest Veneto). The study was carried out according to the Declaration of Helsinki. Informed consent was obtained.

Patients remained in the sitting position (i.e., 90° of hip flexion, knee flexion, and ankle–foot angle) during the evaluation. Upper limb spasticity postures were evaluated and recorded for the shoulder (adducted/internally rotated), elbow (flexed/extended), forearm (pronated/supinated/neutral), wrist (flexed/extended/neutral), and hand (fingers flexed/thumb in palm). An expert physiatrist (more than 10 years of experience in spasticity clinics) evaluated all patients.

The following information was collected by means of chart review: gender, age, disease duration, Expanded Disability Status Scale score (EDSS) (11), Ashworth scale score for the shoulder, elbow, forearm, wrist, and hand. The EDSS was used to assesses the level of disability in people with MS considering some functional systems (pyramidal – weakness or difficulty moving limbs; cerebellar – ataxia, loss of coordination, or tremor; brainstem – problems with speech, swallowing, and nystagmus; sensory – numbness or loss of sensations; bowel and bladder function; visual function; cerebral or mental functions; other) by means of a score ranging from 0 (no disability) to 10 (death). The Ashworth scale was used to assess upper limb spasticity on a 5-point scale for grading the resistance of a relaxed limb to rapid passive stretch (0, no increase in muscle tone; 1, slight increase in muscle tone at the end of the range of motion; 2, more marked increase in muscle tone through most of the range of motion; 3, considerable increase in muscle tone; 4, joint is rigid) (12). As for the previous BoNT-A treatment, injected muscles and dosages were also recorded.

Statistical analysis was carried out using the Statistical Package for the Social Sciences for Macintosh, version 26.0 (IBM Corp, Armonk, NY, USA). Descriptive statistics were used for the demographic and clinical features of our sample (for description purposes BoNT-A dosage was reported in generic units considering a conversion rate of 1:3 for OnabotulinumtoxinA and AbobotulinumtoxinA as well as a conversion rate of 1:1 for OnabotulinumtoxinA and IncobotulinumtoxinA) (13). Frequency analysis was performed to report on the upper limb spasticity postures and patterns.

## RESULTS

Twenty-five patients with MS, screened for the project *2022/R-Single/031* from August 2023 to January 2024, have been included. Given that 9 patients exhibited bilateral upper limb spasticity, we decided to report on a total of 34 upper limbs. The patients’ demographic and clinical features are reported in Table I.

**Table I:** Demographic and clinical features of patients

Age, years, mean (SD)	58.5 (7.6)
Gender, *n*	
Male	12
Female	13
Disease duration, years, mean (SD)	19.4 (9.6)
Type of multiple sclerosis, *n*	
RR	6
SP	11
PP	8
EDSS score, median (IQR)	7 (6; 8)

SD: standard deviation; RR: relapsing-remitting; SP: secondary progressive; PP: primary progressive; EDSS: Expanded Disability Status Scale; IQR: interquartile range.

**Fig. 1:**
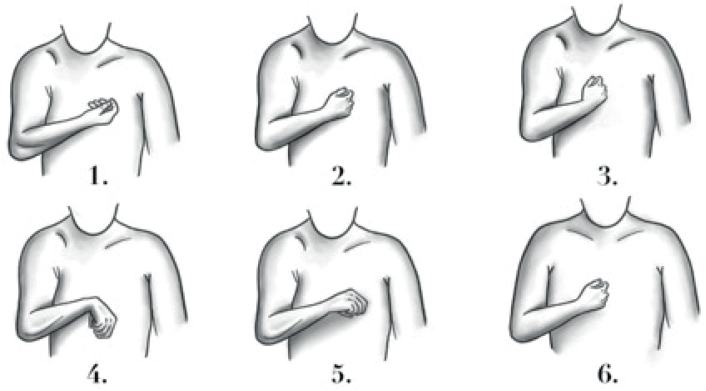
Upper limb spasticity patterns.

Based on our clinical observations, 6 patterns of upper limb spasticity have been described according to the postures of the shoulder, elbow, forearm, and wrist. These patterns are illustrated in Fig. 1.

*Pattern 1* (frequency 17.6%) is characterized by adduction and internal rotation of the shoulder, flexed elbow, supinated forearm, and neutral position of the wrist. *Pattern 2* (frequency 23.5%) is characterized by adduction and internal rotation of the shoulder, flexed elbow, forearm, and wrist in neutral position. *Pattern 3* (frequency 5.9%) is characterized by adduction of the shoulder, flexed elbow, neutral position of the forearm, and flexed wrist. *Pattern 4* (frequency 8.8%) is characterized by adduction and internal rotation of the shoulder, flexed elbow, pronated forearm, and flexed wrist. *Pattern 5* (14.7%) is characterized by adduction of the shoulder, flexed elbow, pronated forearm, and wrist in neutral position. *Pattern 6* (29.5%) is characterized by internal rotation of the shoulder, flexed elbow, forearm, and wrist in neutral position. Regarding the posture of the hand, we did not observe a specific presentation for each of the patterns described above. Information concerning botulinum toxin treatment is reported in Table II.

**Table II:** Treatment features of upper limb spasticity patterns

Pattern 1	
Frequency, %	17.6
Mean BoNT-A dosage (U)	300.9
Pattern 2	
Frequency, %	23.5
Mean BoNT-A dosage (U)	360.9
Pattern 3	
Frequency, %	5.9
Mean BoNT-A dosage (U)	375.9
Pattern 4	
Frequency, %	8.8
Mean BoNT-A dosage (U)	485.9
Pattern 5	
Frequency, %	14.7
Mean BoNT-A dosage (U)	501.9
Pattern 6	
Frequency, %	29.5
Mean BoNT-A dosage (U)	282.7

U: units.

## DISCUSSION

Consistent with the main aim of this pilot study, we provided a classification consisting of 6 upper limb patterns based on various postures involving the shoulder, elbow, forearm, and wrist in patients with MS suffering from upper limb spasticity. Interestingly, our classification has some differences as well as some points in common with the one proposed by Hefter, who described 5 patterns of upper limb spasticity in stroke patients (9). In particular, only 2 of the patterns we observed in patients with MS (namely, *pattern 2* and *pattern 4*) are similar to those described by Hefter in stroke patients (namely, pattern III and pattern IV respectively). Thus, the 2 classifications have only a partial overlap. From an epidemiological standpoint, in patients with MS suffering from upper limb spasticity, *pattern 6* was the most frequent (29.5% of cases) followed by *pattern 2*, which had a frequency of 23.5%. Conversely, in Hefter’s classification, pattern III (characterized by internal rotation and adduction of the shoulder, flexed elbow, forearm, and wrist in neutral position) was the most frequent (41.8% of cases) followed by pattern I (characterized by internal rotation and adduction of the shoulder, flexed elbow, supinated forearm, and flexed wrist), which had a frequency of 24.8% in patients with post-stroke spasticity (9). Considering postures (i.e., postural attitude of a single joint/anatomical region), it is noteworthy that none of our patients presented with extended joints. This contrasts with Hefter’s observations, which included 2 patterns (namely, II and V) with extended postures of the wrist and elbow, respectively. Unfortunately, we did not find specific postures of the hand related to the upper limb spasticity patterns shown in Fig. 1. This aligns with Hefter and colleagues’ classification, which specified that any spastic hand posture could be combined with all upper limb spasticity patterns (9). A recent paper reported on a Canadian cross-sectional survey involving 50 physiatrists who treat patients with stroke, MS, cerebral palsy, brain injury, and spinal cord injury in their clinical practice (14). Using Hefter’s classification system, upper limb spasticity patterns from all aetiologies were ranked in the following order from most to least common: patterns IV > I > III > V > II. This differs slightly from the frequency reported by Hefter and colleagues in their original paper, where upper limb post-stroke spasticity patterns were ranked as follows: III > I > IV > II > V (9). Consistently, although all 5 of Hefter’s postures were still observed in spasticity clinical practice (14), the differing frequencies between stroke alone and stroke combined with other aetiologies (e.g., MS, cerebral palsy, brain injury, and spinal cord injury) underscore the need to develop a classification system specifically for upper limb spasticity caused by conditions other than stroke.

Spasticity is a positive symptom of the upper motor neuron syndrome. So, it is not surprising to observe different features of spasticity depending on its various aetiologies (e.g., stroke, MS, brain injury, spinal cord injury). In particular, for patients with MS, specific spasticity patterns (i.e., sets of limb postures) may be related to the heterogenicity of demyelination lesion locations and the clinical course of disease (13). In our sample, patients with *pattern 5* (i.e., adduction of the shoulder, flexed elbow, pronated forearm, and wrist in neutral position) received the highest mean dose of BoNT-A (501.9 units), while those with *pattern 6* (internal rotation of the shoulder, flexed elbow, forearm, and wrist in neutral position) and *pattern 1* (i.e., adduction and internal rotation of the shoulder, flexed elbow, supinated forearm, and wrist in neutral position) received lower mean doses of BoNT-A (282.7 and 300.9 units, respectively). Interestingly, the dosages of BoNT-A administered to our patients with MS are quite similar to those suggested for post-stroke spasticity (15). This might seem unusual given that previous literature has identified different features of spasticity in MS versus stroke patients, in terms of both neurological and rheological (non-neurological) components (16). The administration of BoNT-A is guided by product labelling that specifies approved dosages per posture and muscle, without clearly differentiating among all aetiologies of spasticity. This might explain why our findings align with the current literature, despite the clinical bias that similar spasticity patterns may arise from different pathologies (10). Additionally, our patients exhibited a high level of disability (median EDSS 7) compared with other studies (17). High doses of BoNT-A can result in relevant (though not permanent) muscle paralysis, while lower doses may allow some muscle movement. This could partly explain why our patients received doses comparable to those used in post-stroke spasticity, which are typically higher than those used for patients with MS (10, 18).

This pilot study has several limitations. First, the sample size is small (e.g., *pattern 3* was observed in only 2 limbs, and *pattern 4* in only 3 subjects). Consequently, we can draw only preliminary rather than definitive conclusions. Although the sample size was limited (i.e., 25 patients and 34 upper limbs), it is consistent with the pilot nature of this study, current literature, and the epidemiology of spasticity in MS, where upper limb involvement is less frequent compared with the lower limbs (1, 6). Second, only 1 clinical assessor evaluated all patients. To reduce subjectivity, independent evaluations by 2 or more raters, potentially using photos or videos, would be preferable. Third, our study relied solely on clinical evaluations and did not incorporate instrumental assessments such as polyelectromyography or motion analysis. However, given the high level of disability in our sample (see Table I), there was little likelihood of relevant active function in the limbs. Therefore, the role of instrumental analysis is limited.

In conclusion, this observational pilot study identified 6 patterns of upper limb spasticity in patients with MS, which show only partial overlap with those observed in patients with post-stroke spasticity. This supports the need to consider the specific features of spasticity related to its aetiology when managing patients with upper motor neurone syndrome. Future, larger prospective studies are needed to address this issue, taking into account and overcoming the limitations of this pilot study.
